# Concordance Index-Based Comparison of Inflammatory and Classical Prognostic Markers in Untreated Hepatocellular Carcinoma

**DOI:** 10.3390/jcm14155514

**Published:** 2025-08-05

**Authors:** Natalia Afonso-Luis, Irene Monescillo-Martín, Joaquín Marchena-Gómez, Pau Plá-Sánchez, Francisco Cruz-Benavides, Carmen Rosa Hernández-Socorro

**Affiliations:** 1 Hepato-Bilio-Pancreatic Surgery Unit, Department of General and Digestive Surgery, Hospital Universitario de Gran Canaria Dr. Negrín, Bco. La Ballena s/n, 35012 Las Palmas de Gran Canaria, Spain; nafolui@gobiernodecanarias.org (N.A.-L.); paups88@gmail.com (P.P.-S.); fcruben@gobiernodecanarias.org (F.C.-B.); 2 Facultad de Ciencias de la Salud, Universidad de Las Palmas de Gran Canaria, 35001 Las Palmas de Gran Canaria, Spain; imonmarf@gobiernodecanarias.org (I.M.-M.); carmenrosa.hernandez@ulpgc.es (C.R.H.-S.); 3 Department of Radiology, Hospital Universitario de Gran Canaria Dr. Negrín, Bco. La Ballena s/n, 35012 Las Palmas de Gran Canaria, Spain

**Keywords:** hepatocarcinoma, prognostic factors, neutrophil-to-lymphocyte ratio

## Abstract

**Background/Objectives**: Inflammation-based markers have emerged as potential prognostic tools in hepatocellular carcinoma (HCC), but comparative data with classical prognostic factors in untreated HCC are limited. This study aimed to evaluate and compare the prognostic performance of inflammatory and conventional markers using Harrell’s concordance index (C-index). **Methods**: This retrospective study included 250 patients with untreated HCC. Prognostic variables included age, BCLC stage, Child–Pugh classification, Milan criteria, MELD score, AFP, albumin, Charlson comorbidity index, and the inflammation-based markers neutrophil-to-lymphocyte ratio (NLR), platelet-to-lymphocyte ratio (PLR), monocyte-to-lymphocyte ratio (MLR), Systemic Inflammation Response Index (SIRI), and Systemic Immune-inflammation Index (SIII). Survival was analyzed using Cox regression. Predictive performance was assessed using the C-index, Akaike Information Criterion (AIC), and likelihood ratio tests. **Results**: Among the classical markers, BCLC showed the highest predictive performance (C-index: 0.717), while NLR ranked highest among the inflammatory markers (C-index: 0.640), above the MELD score and Milan criteria. In multivariate analysis, NLR ≥ 2.3 remained an independent predictor of overall survival (HR: 1.787; 95% CI: 1.264–2.527; *p* < 0.001), along with BCLC stage, albumin, Charlson index, and Milan criteria. Including NLR in the model modestly improved the C-index (from 0.781 to 0.794) but significantly improved model fit (Δ–2LL = 10.75; *p* = 0.001; lower AIC). **Conclusions**: NLR is an accessible, cost-effective, and independent prognostic marker for overall survival in untreated HCC. It shows discriminative power comparable to or greater than most conventional predictors and may complement classical stratification tools for HCC.

## 1. Introduction

According to GLOBOCAN 2022 estimates, liver cancer accounted for approximately 865,000 new cases and over 750,000 deaths globally, ranking as the third leading cause of cancer-related mortality—following lung and colorectal cancers—and the sixth most commonly diagnosed malignancy [1]. Hepatocellular carcinoma (HCC) represents the predominant histological subtype, comprising 75–85% of all liver cancer cases. Chronic infection with hepatitis B or C virus remains a major etiological factor, contributing to an estimated 21–55% of cases worldwide [2]. Additional risk factors include exposure to aflatoxins, excessive alcohol intake, metabolic conditions such as obesity and diabetes, tobacco use, metabolic dysfunction-associated steatotic liver disease (MASLD), as well as male sex and older age [3]. The relative importance of these risk factors varies by geographic region.

The American Association for the Study of Liver Diseases indicates that cirrhosis, regardless of its etiology, is the main risk factor, and that the epidemiological transition is shifting the burden toward older individuals with metabolic risk factors, although viral hepatitis remains relevant in endemic areas [4]. Moreover, the incidence in women is increasing in relation to MASLD, approaching that of men within this subgroup, although the overall burden remains higher in males [5]. Of note is that the incidence of hepatocellular carcinoma related to MASLD is increasing, especially in Western countries, and is now considered one of the leading non-viral causes of HCC [6].

Although it has improved in recent decades, the 5-year overall survival rate for patients still remains around 22% [7].

The prognosis of patients with hepatocellular carcinoma is determined by tumor-related characteristics, liver function, the patient’s general condition, and serum biomarkers [8,9]. Among these, the most relevant and consistently reported factors in the literature include the following: advanced age, male sex, and alcoholic liver disease [10]; the Barcelona Clinic Liver Cancer (BCLC) staging system [11]; the Child–Pugh classification, the Model for End-Stage Liver Disease (MELD) score [12]; elevated serum alpha-fetoprotein (AFP) levels [13]; the Milan criteria [14]; and albumin [15].

It is worth noting that, despite the recent publication of several studies on the subject [16,17,18,19], inflammatory biomarkers are not commonly included in the classical lists of prognostic factors for hepatocellular carcinoma [8,14,20] or are only briefly mentioned [21]. Nevertheless, there is increasing evidence that the presence of systemic inflammation in cancer patients, including those with HCC, is associated with poorer prognosis in terms of disease progression and survival [22,23].

In this sense, several hematological parameters derived from a simple peripheral blood sample have been proposed as inflammatory prognostic markers: neutrophil-to-lymphocyte ratio (NLR) [24], platelet-to-lymphocyte ratio (PLR) [25], monocyte-to-lymphocyte ratio (MLR) [26], serum C-reactive protein [27], C-reactive protein-to-albumin ratio [28], Systemic Inflammation Response Index (SIRI) [29], Systemic Immune-inflammation Index (SIII) [30], and other more complex scales, such as the modified Glasgow Prognostic Score [31]. Recent studies have highlighted that these biomarkers may be helpful in revealing the underlying inflammatory state. Moreover, most of them, to a lesser or greater degree, have been reported as good prognostic markers in a large number of neoplasms, including untreated [13,16,32,33,34,35] and treated HCC [36,37].

However, few studies have directly compared the prognostic utility of inflammatory biomarkers, either against one another or in relation to established prognostic factors, in patients with hepatocellular carcinoma. This study aimed to evaluate and compare the prognostic value for overall survival of the most accessible inflammatory markers (NLR, PLR, MLR, SIRI, and SII), adjusting for conventional prognostic variables as potential confounders in a well-defined cohort of patients with untreated HCC.

## 2. Materials and Methods

### 2.1. Design and Study Population

An observational retrospective study was conducted on a cohort of 250 consecutively included patients diagnosed with hepatocellular carcinoma between 2011 and 2020 in our institution. The setting was a tertiary care hospital that served a population of approximately 400,000 inhabitants. From an initial cohort of 297 patients, a total of 47 individuals were excluded due to incomplete follow-up or missing essential clinical data. No patients were excluded based on disease stage, liver function, treatment intent, or baseline performance status. The most frequent reason for exclusion was loss to follow-up, which occurred in 36 patients who had been referred from three peripheral centers not integrated into our institutional electronic health records. In the remaining 11 excluded cases, follow-up was not possible due to individual circumstances, such as voluntary treatment discontinuation or transfer to other healthcare regions. The characteristics of these excluded patients were not collected.

### 2.2. Management of the Patients

All patients included in this study were diagnosed with HCC through one or two dynamic imaging radiological tests (liver contrast-enhanced ultrasound, contrast-enhanced computed tomography and/or Magnetic Resonance Imaging with contrast) or a clear histological or cytological diagnosis of hepatocellular carcinoma. Histological samples were obtained by means of percutaneous fine needle aspiration cytology, core needle biopsy, and/or resection specimens.

Once the diagnosis was established, staging was determined according to the Barcelona Clinic Liver Cancer (BCLC) classification [11]. Following these criteria, treatment was then proposed. The early stages of BCLC 0-A were treated with curative intent by liver resection, ablation, or liver transplantation, while in the other more advanced stages the intent was no longer curative and treatment was applied by transcatheter arterial chemoembolization (TACE), systemic treatment, or simply palliative measures.

### 2.3. Follow-Up

The patients were entered into the study with the date determined as the day on which the first definitive treatment was applied after diagnosis. All of them were followed up by periodic visits to the hospital outpatient clinic. In the long term, it was possible to determine whether the patient was still alive or had died and the date of exit by consulting the hospital and/or primary care databases. The overall mean follow-up time for the entire cohort was 20.4 months, while for surviving patients it was 48.4 months.

### 2.4. Study Variables

Clinical data were extracted from patients’ medical records, entered into a database, and subsequently analyzed. The following variables were evaluated:

*Baseline characteristics.* Age and sex at the time of the diagnosis.

*Comorbidity*. The Charlson Comorbidity Index was calculated for each patient. This index includes 19 medical conditions, each weighted from 1 to 6 points, with total scores ranging from 0 to 37, depending on the presence and severity of comorbid diseases. A score of 0 indicates the absence of comorbidities. Generally, scores of 1–2 indicate low comorbidity, 3–4 moderate comorbidity, and scores greater than 4 indicate high comorbidity [38]. In this study, the index was not adjusted for age or for the presence of AIDS, as no patients in the cohort had this condition.

*Assessment of Underlying Liver Disease.* The etiology of liver disease (no underlying liver disease, alcohol-related cirrhosis, viral cirrhosis HBV/HCV, and non-alcoholic/non viral cirrhosis, including MASLD and other etiologies), Child–Pugh classification, and the MELD score [39] were determined and recorded.

*Laboratory Data*. At the time of diagnosis, the following laboratory values were recorded: absolute neutrophil, lymphocyte, monocyte, and platelet counts; and alpha-fetoprotein (AFP) levels.

*Inflammation-Based Prognostic Markers (Pretreatment).* The neutrophil-to-lymphocyte ratio was calculated by dividing the absolute neutrophil count by the absolute lymphocyte count (N/L). The platelet-to-lymphocyte ratio (PLR) was obtained by dividing the platelet count by the lymphocyte count (P/L). The monocyte-to-lymphocyte ratio (MLR) was calculated as M/L. The Systemic Inflammation Response Index (SIRI) was defined as the product of the neutrophil and monocyte counts divided by the lymphocyte count (N × M/L) [29]. The Systemic Immune-Inflammation Index (SIII) was calculated by multiplying the neutrophil and platelet counts and dividing the result by the lymphocyte count (N × P/L) [30].

*Conventional Prognostic Factors.* Patients were separated into five groups (0, A, B, C, or D) according to the Barcelona Clinic Liver Cancer classification [11]. Additional prognostic factors included the Milan criteria [40], serum albumin levels (g/dL), and serum AFP levels (ng/mL).

*Therapeutic Options.* All treatment modalities received by the patients were documented. These included ablation therapy (percutaneous ethanol injection, radiofrequency ablation, or microwave ablation), transarterial chemoembolization (TACE), surgical resection, liver transplantation, systemic therapy, and/or palliative care measures.

*Outcome Variable.* Long-term survival was considered the primary outcome. Survival time was defined as the interval between the date of inclusion in the study (i.e., the date of initial treatment) and the date of death, regardless of whether the cause of death was related to the neoplasm.

### 2.5. Statistical Analysis

The data were entered and analyzed using SPSS statistical package v. 29.0 (IBM Corporation, Armonk, NY, USA) and the RStudio software (v. 4.5.1). Figures were created using the software Jamovi (v. 2.6.26).

*Descriptive analysis.* A descriptive analysis of the study sample was conducted. Categorical variables were summarized using frequencies and percentages. For numerical variables, results were expressed as mean ± standard deviation or median with interquartile range, depending on the distribution of the data. The Kolmogorov–Smirnov test was used to assess the normality of distributions. Survival analysis was performed using the Kaplan–Meier method.

*Univariate analysis.* A univariate Cox proportional hazards regression was conducted to evaluate the association between each predictor variable, including inflammatory markers, and overall survival. The Log-Rank test was used to compare survival curves.

Subsequently, the discriminative ability of each classical prognostic factor for hepatocellular carcinoma, along with the inflammatory markers, was assessed. For this purpose, Harrell’s concordance index (C-index) [41,42] was calculated using univariate Cox regression models. The C-index reflects the probability that, for a random pair of patients, the one with the worse predicted prognosis according to the model will indeed die before the other. C-indices were reported along with their standard errors and 95% confidence intervals using the “rcorr.cens()” function from the Hmisc package in R. This approach also allowed for the identification of the most accurate survival predictor among the inflammatory markers. The C-index ranges from 0.5 (no predictive ability) to 1.0 (perfect discrimination), with higher values indicating superior performance in distinguishing between patients with different survival outcomes.

Given the pronounced skewness observed in the distribution of the numerical variables, these were analyzed as categorical variables using specific cutoff values. The cutoffs applied were as follows: 2.3 for NLR, 100.0 for PLR, 0.30 for MLR, 1.0 for SIRI, and 400.0 for SIII. These thresholds were obtained by rounding the values identified using the Youden index (NLR, PLR, MLR) and by referencing the previously published literature (SIRI [43], SIII [44]). Accordingly, NLR values were categorized as <2.3 or ≥2.3; PLR as <100 or ≥100; MLR as <0.30 or ≥0.30; SIRI as <1.0 or ≥1.0; and SIII as <400 or ≥400 for all subsequent analyses.

*Multivariate analysis and model comparison.* A multivariate Cox proportional hazards regression model was constructed, in which the best survival predictor among the inflammatory markers was adjusted for the classical prognostic factors of hepatocellular carcinoma, including age, Charlson comorbidity index, BCLC classification, Child–Pugh score, Milan criteria, MELD score, serum AFP, and serum albumin levels. The objective was to determine which variables remained independent prognostic factors for overall survival.

To evaluate and compare model performance, the concordance index (C-index), Akaike Information Criterion (AIC), and the likelihood ratio test were calculated for models with and without the inclusion of the NLR.

To assess the model’s overall discriminative ability, the C-index was computed. Two multivariate models were fitted: one including the NLR and another excluding it. The C-indices of both models were compared, and the statistical significance of the difference was evaluated using the z-score formula for differences between independent means, incorporating the pooled standard error.

To evaluate model fit while considering complexity, the AIC was also calculated. A lower AIC value indicates a better balance between goodness-of-fit and parsimony. This metric was used to compare the full model including the NLR, with a reduced model excluding it.

In addition, the likelihood ratio test was performed to determine whether the inclusion of NLR significantly improved the model’s explanatory power. This test is based on the difference in the –2 log-likelihood (–2LL) values between the two models.

The strength of association was expressed using hazard ratios (HRs) with their corresponding 95% confidence intervals (CIs). Each C-index was reported along with its 95% CI and standard error (SE). A two-sided *p*-value of <0.05 was considered statistically significant.

## 3. Results

### 3.1. Baseline Characteristics

Of the 250 patients included in this study, 195 (78%) were men and 55 (22%) were women (*p* < 0.001), resulting in a male-to-female ratio of 3.5:1 (*p* < 0.001). The mean age was 68.0 ± 10.4 years.

The underlying cause of liver disease was alcohol-related cirrhosis in 80 patients (32.0%), viral cirrhosis in 78 (31.2%), and non-alcohol/non-viral cirrhosis in 73 (29.2%), including 12 cases (4.8%) in which the etiology could not be determined. In 19 patients (7.6%), no evidence of underlying liver disease was found.

Regarding the treatment received, ablation was the most frequently used initial therapy with curative intent, performed in 94 patients (37.6%). An additional 27 patients (10.8%) received ablation either as a bridge to liver transplantation or as a second-line treatment. TACE was used as the first-line treatment in 31 patients (12.4%) and as a second-line option in 14 additional cases. Liver transplantation was initially proposed for 35 patients, but only 13 ultimately underwent the procedure. Surgical resection was offered as the initial treatment in 8 patients (3.2%). Systemic therapy was administered as the first-line treatment in 19 patients (7.6%) and as a second- or third-line option in 37 patients (14.8%). In 84 cases (33.7%), patients were referred for palliative care as the initial therapeutic approach.

**Figure 1:**
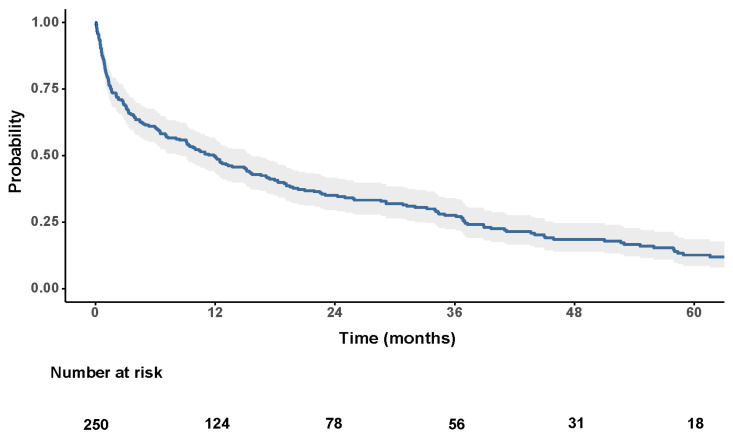
Kaplan–Meier overall survival curve for the entire cohort of patients with hepatocellular carcinoma.

The overall survival curve is presented in Figure 1. At the end of follow-up, only 43 patients (17.2%) were still alive, while the remaining 207 patients (82.8%) had died. Regardless of disease stage and treatment modality, the overall probability of survival at 1, 3, and 5 years was 49.8%, 27.6%, and 12.7%, respectively. The estimated mean survival was 27.1 months (SE: 2.6; 95% CI: 22.0–32.2), and the estimated median survival was 11.9 months (IQR: 1.6–37.0).

### 3.2. Univariate Cox Regression Analysis

**Figure 2:**
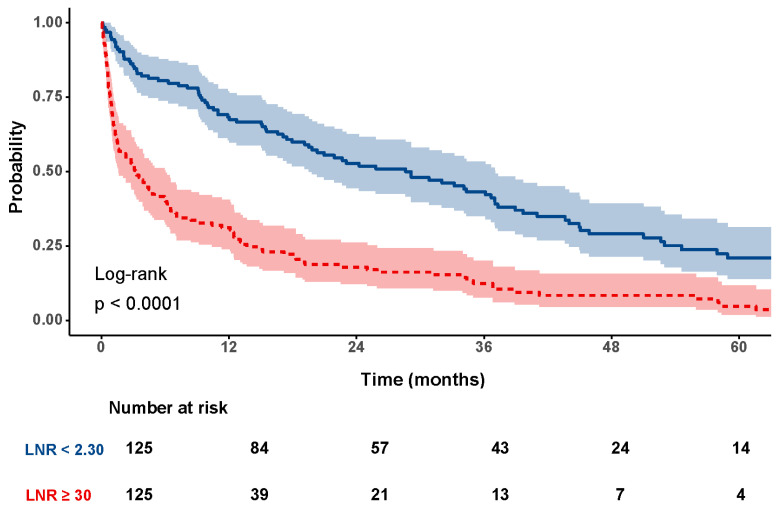
Unadjusted survival curves for patients with baseline neutrophil-to-lymphocyte ratio (NLR) <2.30 (blue) and patients with baseline NLR ≥2.30 (red) (*p* < 0.001).

The baseline characteristics of the study population and the results of the univariate Cox regression analysis for survival are presented in Table 1. Figure 2 displays the unadjusted Kaplan–Meier survival curves according to baseline NLR levels. Patients with elevated NLR values had an estimated 5-year survival probability of 4.8%, compared to 21.0% in those with NLR values below 2.30 (*p* < 0.001).

### 3.3. C-Index-Based Evaluation of Prognostic Variables

The individual C-index values for each prognostic variable are presented in Table 2. According to this index, the three strongest predictors of survival in untreated hepatocellular carcinoma were the BCLC classification (C-index: 0.717; SE: 0.017; 95% CI: 0.750–0.864), serum albumin level (C-index: 0.713; SE: 0.020; 95% CI: 0.674–0.752), and Child–Pugh Score (C-index: 0.677; SE: 0.018; 95% CI: 0.642–0.712. Among the inflammatory markers, the NLR was the best predictor (C-index: 0.640; SE: 0.016; 95% CI: 0.609–0.671), ranking fifth overall, above the Milan criteria and the MELD score.

### 3.4. Multivariate Cox Regression Analysis

**Figure 3:**
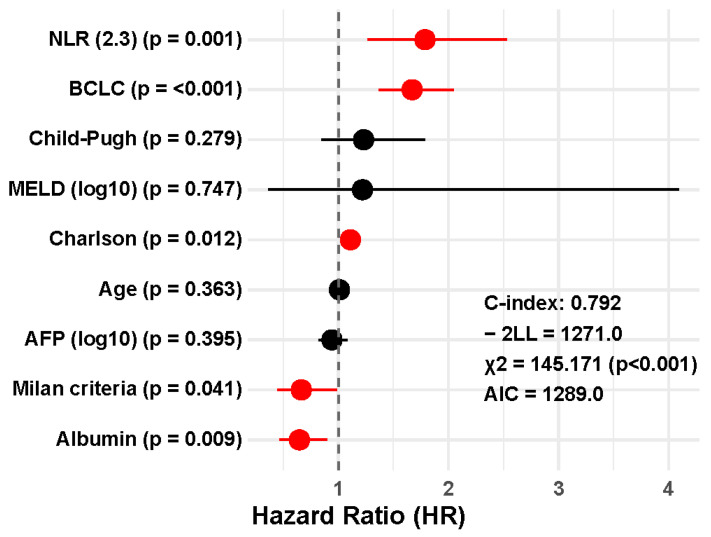
Forest plot of the multivariate Cox regression model including the neutrophil-to-lymphocyte ratio (NLR) as a covariate for overall survival in patients with untreated hepatocellular carcinoma.

In the multivariate Cox regression analysis (Figure 3), the inflammatory marker NLR (≥2.3) remained an independent prognostic factor for overall survival in patients with untreated hepatocellular carcinoma, with a hazard ratio (HR) of 1.787 (95% CI: 1.264–2.527; *p* < 0.001). This association persisted after adjustment for classical prognostic variables, including BCLC stage (HR: 1.670; *p* < 0.001), serum albumin level (HR: 0.647; *p* = 0.009), Charlson comorbidity index (HR: 1.110; *p* = 0.012), and Milan criteria (HR: 0.664; *p* = 0.041). In contrast, age, Child–Pugh classification, AFP level, and MELD score did not reach statistical significance in the model. The overall model test was significant (χ^2^ = 145.171; df = 9; *p* < 0.001), indicating good overall model fit.

**Figure 4:**
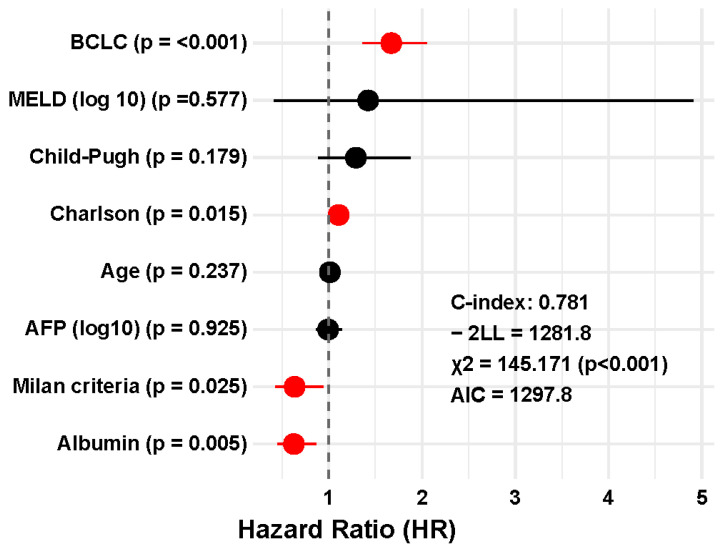
Forest plot of the multivariate Cox regression model excluding the neutrophil-to-lymphocyte ratio (NLR) for overall survival in patients with untreated hepatocellular carcinoma.

The multivariable model that included the NLR (Figure 3) yielded a C-index of 0.792 (SE: 0.017; 95% CI: 0.759–0.825), whereas the model excluding NLR (Figure 4) showed a C-index of 0.781 (SE: 0.018; 95% CI: 0.746–0.816). Although the absolute C-index was slightly higher when the inflammatory marker was included, the difference between the two models did not reach statistical significance (z = 0.44; *p* = 0.658).

However, the comparison of the –2 log-likelihood (–2LL) values revealed a significant improvement in model fit. The model including NLR had a –2LL of 1271.0, compared to 1281.8 for the model without NLR, resulting in a statistically significant difference in log-likelihood (Δ–2LL = 10.75; *p* = 0.001).

Finally, the full multivariable Cox model that included NLR also demonstrated superior fit based on the Akaike Information Criterion (AIC). The AIC for the model with NLR was 1289.0, compared to 1297.8 for the model without it. This difference supports the inclusion of NLR as a relevant prognostic factor, reflecting an improved balance between model complexity and explanatory power.

No collinearity was observed among covariates in either model, and all generalized variance inflation factors remained below 1.5.

## 4. Discussion

Hepatocellular carcinoma remains a major global health concern due to its high incidence and poor prognosis. Despite advances in diagnosis and treatment, survival rates remain suboptimal, particularly in patients with advanced-stage or unresectable disease. Prognostic stratification plays a pivotal role in guiding treatment decisions and predicting outcomes. While classical prognostic systems such as BCLC, MELD score, and Child–Pugh are well established, there is growing interest in identifying accessible, inexpensive, and biologically relevant markers that reflect tumor biology and systemic host response, particularly inflammation, which is increasingly recognized as a key component in cancer progression.

This study demonstrates that assessing the inflammatory status of patients with untreated hepatocellular carcinoma at the time of diagnosis may serve as a valuable tool for predicting overall survival. Among all the inflammatory markers evaluated in this cohort, the neutrophil-to-lymphocyte ratio emerged as the strongest predictor of survival.

In the multivariate analysis, NLR remained an independent prognostic factor after adjustment for classical non-inflammatory prognostic variables. Regarding its discriminative capacity, NLR ranked fifth according to Harrell’s concordance index (C-index) when compared to both inflammatory and non-inflammatory markers, surpassing even the MELD score and the Milan criteria. These findings position NLR as a relevant prognostic marker, with greater discriminative power than several classical parameters commonly used in the stratification of hepatocellular carcinoma.

Moreover, although the inclusion of NLR in the Cox regression model was associated with a modest increase in the C-index, it led to a statistically significant improvement in overall model performance. These results suggest that NLR provides independent prognostic value and enhances the explanatory capacity of the model, even if its contribution to individual-level discrimination is limited.

However, although statistically supported by improvements in –2LL and AIC, such incremental gains may not always translate into clear clinical benefit. Therefore, the prognostic utility of NLR should be interpreted with caution and ideally confirmed through prospective validation in larger and more diverse cohorts.

The role of neutrophils in the progression of solid tumors, including hepatocellular carcinoma, and their potential as therapeutic targets has been widely studied [23]. HCC is recognized as an inflammation-related cancer in which non-resolving inflammation contributes to both tumor development and progression. Neutrophils play a central role in shaping the immune microenvironment of these tumors. A high density of intratumoral neutrophils, commonly referred to as tumor-associated neutrophils (TANs) [45], has been identified within the tumor microenvironment. Elevated TAN infiltration has been associated with worse recurrence-free, cancer-specific, and overall survival. Moreover, TANs may exert both anti-tumorigenic and pro-tumorigenic effects, a duality that carries important therapeutic implications [46]. Within this context, the NLR may serve as a useful prognostic indicator of tumor progression.

Several previously published studies have examined the prognostic significance of inflammatory markers in patients with hepatocellular carcinoma [35].

Regarding NLR, most studies have demonstrated a significant association with overall survival in univariate analyses [33,47,48,49,50,51,52,53,54]. However, only a few of them [47,49] identified NLR as an independent prognostic factor in multivariate models. In some cohorts, this association could not be confirmed [55], likely due to limited sample size. Analyses using ROC curves have yielded inconsistent results: while some studies reported excellent areas under the curve (AUC) [54], others produced paradoxical findings [56]. Nevertheless, several meta-analyses [16,17,19,34,57,58] have concluded that NLR is clearly an effective prognostic factor in patients with HCC, particularly among East Asian populations, where the incidence of the disease is higher.

These studies appear to exhibit considerable heterogeneity in the cutoff values used to define elevated NLR, which complicates standardization and direct comparison of results across different cohorts. Indeed, in the reviewed series, cutoff values ranged from 1.7 [52] to 4.2 [53]. In our study, a cutoff of 2.3 was applied. Nevertheless, regardless of the specific threshold used, most studies—including several meta-analyses—have found a statistically significant association between elevated NLR and reduced overall survival.

Regarding other inflammatory markers, several studies, including three meta-analyses [18,59,60,61], have demonstrated that elevated platelet-to-lymphocyte ratio (PLR) is independently associated with poorer overall survival and a higher risk of recurrence in patients with HCC [48,49,51,52,53,62]. Both the monocyte-to-lymphocyte ratio (MLR) and the lymphocyte-to-monocyte ratio (LMR) have also been evaluated as prognostic factors in HCC patients. Some studies have shown that elevated preoperative MLR levels are associated with worse survival outcomes [52,63]. Conversely, patients with higher preoperative LMR values appear to have a better prognosis [48,64,65]. However, other series have failed to confirm the prognostic utility of these markers in HCC [53].

A well-established association has also been observed between the Systemic Inflammation Response Index (SIRI) and the prognosis of untreated hepatocellular carcinoma. Several studies and meta-analyses have shown that elevated SIRI values [43,66], as well as other systemic inflammation indices such as the Systemic Immune-inflammation Index (SIII) [67], are independently associated with worse overall survival and increased tumor aggressiveness in patients with hepatocellular carcinoma, regardless of treatment status, even in advanced-stage disease without specific therapy [68,69].

Finally, other inflammatory markers such as serum C-reactive protein (CRP) [32], the C-reactive protein-to-albumin ratio [70], the gamma-glutamyl transpeptidase-to-platelet ratio (GPR) [50], and the aspartate aminotransferase-to-lymphocyte ratio (ALR) [51], have also been linked to survival, although they were not included in the present study.

It is important to emphasize that the magnitude of a variable’s association with survival, typically expressed as the hazard ratio in most studies, is not equivalent to its individual predictive power, either alone or within the context of a regression model. In such cases, the concordance index (C-index) is more commonly used. In survival analysis, the hazard ratio quantifies the effect of a predictor on the risk of the event occurring, indicating how much the hazard changes with each unit increase in the predictor [71]. The C-index, on the other hand, measures the model’s discriminatory ability by estimating the proportion of correctly ordered patient pairs based on predicted survival times. While the HR provides insight into the strength and direction of associations, the C-index evaluates the overall predictive accuracy of the model in distinguishing survival outcomes [72].

We acknowledge that other approaches, such as time-dependent ROC curves and Net Reclassification Index, could complement these findings. These methods may be particularly helpful in dynamic or individualized risk prediction models and should be considered in future prospective validation studies.

In fact, several studies have employed receiver operating characteristic (ROC) curves to evaluate the prognostic utility of inflammatory markers in relation to hepatocellular carcinoma [51,53,54,56]. However, the reported areas under the curve ranged from 0.479 [53] to 0.811 [54], generally indicating a poor performance, except for the latter series. In our study, we did not use ROC curve analysis, as it treats mortality as a binary outcome rather than accounting for survival time. This limitation may lead to an underestimation of the true prognostic value of the variables.

As in the previously mentioned studies, all inflammatory markers analyzed in our cohort were significantly associated with overall survival in the univariate analysis. The same was observed for the classical prognostic variables included in the analysis. However, when evaluating the predictive power of each variable using the concordance index (C-index), only a few surpassed the threshold of 0.6. These included the MELD score, Milan criteria, NLR, Charlson comorbidity index, serum albumin, and BCLC classification. Among all variables, the BCLC classification showed the highest prognostic accuracy for untreated hepatocellular carcinoma, with a concordance index (C-index) of 0.717. Among the inflammatory markers, NLR yielded the highest C-index (0.640). The remaining inflammatory markers—PLR, MLR, SIRI, and SIII—showed individual predictive power below 0.6 according to the C-index. For this reason, only NLR was included as the representative inflammatory marker in the multivariate analysis. Assessing whether the low predictive performance observed for the other inflammatory markers was due to the selection of cutoff values for the numerical variables was beyond the scope of this study.

In addition to its statistical significance in the multivariate Cox model, the inclusion of NLR improved the overall model fit, as evidenced by a reduction in AIC. While the increase in discriminative ability (as measured by the concordance index) was modest and did not reach statistical significance, the AIC reflected a more favorable trade-off between model complexity and explanatory strength. Moreover, the inclusion of NLR in the multivariable Cox regression model led to a significant improvement in model fit, with a significant reduction in –2 log-likelihood. This statistically significant change indicates that the model including NLR better captures survival-related variability in untreated hepatocellular carcinoma.

These findings support the utility of NLR as an easily accessible prognostic marker, with predictive power potentially comparable to that of classical tools used in the prognostic stratification of hepatocellular carcinoma. This parameter should be routinely included in standard hematological reports and considered among the general prognostic factors for patients with hepatocellular carcinoma.

A key strength of this study lies in the comparative analytical approach, which incorporates Harrell’s concordance index (C-index), the Akaike Information Criterion (AIC), and the likelihood ratio test. This multifaceted methodology provides a more nuanced and comprehensive evaluation of the predictive performance of individual variables, complementing traditional estimates based solely on hazard ratios.

However, a key limitation of this study is its retrospective, single-center design, which may restrict the generalizability of our findings. Furthermore, a large number of patients were excluded from the initial database due to lack of follow-up or incomplete data, which could introduce selection bias. Most of these were referred from peripheral hospitals lacking data integration.

On the other hand, the prognostic performance of the NLR and the associated C-index improvements reported here require external validation in independent, multicenter cohorts. Future studies should be encouraged to include validation cohorts.

Another limitation is the absence of a competing risks analysis. Although our primary outcome was overall survival, defined irrespective of the cause of death, it is possible that a proportion of deaths in this elderly and comorbid cohort were unrelated to HCC. Future studies should consider incorporating competing risk models to better differentiate between cancer-specific and non-cancer mortality and to refine the prognostic role of inflammatory markers in this context.

The potential for bias must also be acknowledged due to the exclusion of other possible confounding factors related to hepatocellular carcinoma prognosis, particularly in the molecular and genetic domains. Nevertheless, we believe that these findings are highly applicable to routine clinical practice in the management of patients with hepatocellular carcinoma.

## 5. Conclusions

The NLR may be considered as one of the most relevant prognostic markers for overall survival in patients with untreated hepatocellular carcinoma. Given its simplicity, accessibility, and clinical relevance, NLR should be integrated into prognostic assessments for hepatocellular carcinoma.

In our study, it demonstrated a discriminative capacity comparable to or even greater than several classical predictors, such as the MELD score or Milan criteria. From a clinical perspective, NLR could contribute to refining prognostic models and may support therapeutic decision-making, particularly in settings where more complex biomarkers are unavailable.

Future studies are warranted to validate these findings in larger, multicenter cohorts and to explore the added value of combining NLR with molecular and genetic markers for more refined risk stratification.

## Figures and Tables

**Table 1:** Baseline characteristics of the study population and results of the univariate Cox regression analysis for overall survival.

Variable	*n* (%)	Univariate Analysis (HR–CI 95%)	*p*
Age (mean ± SD)	68 (±10.4)	1.03 (1.01–1.05)	<0.001 *
Sex:		0.91 (0.65–1.26)	0.561
Men	195 (78.0)		
Women	55 (22.0)		
Charlson score (median–IQR)	7 (6.0–9.0)	1.27 (1.20–1.34)	<0.001 *
Diabetes mellitus:		1.16 (0.89–1.53)	0.277
No	133 (53.2)		
Yes	117 (46.8)		
Underlying etiology:		0.96 (0.83–1.12)	0.632
No hepatopathy	19 (7.6)		
OH cirrhosis	80 (32.0)		
Viral cirrhosis	78 (31.2)		
Non-OH/non-viral cirrhosis	73 (29.2)		
AFP (Log_10_) (ng/mL) (median–IQR)	10.27 (3.15–428.46)	1.33 (1.19–1.48)	<0.001 *
Albumin (g/dL) (median–IQR)	3.4 (2.8–4.0)	0.46 (0.37–0.56)	<0.001 *
Child–Pugh classification:		2.49 (2.01–3.08)	<0.001 *
A	127(50.8)		
B	75 (30.0)		
C	26 (10.4)		
Milan criteria:		0.36 (0.26–0.48)	<0.001 *
No	105 (42.0)		
Yes	143 (57.2)		
BCLC classification:		2.10 (1.93–2.40)	<0.001 *
0	11 (4.4)		
A	101 (40.4)		
B	49 (19.6)		
C	57 (22.8)		
D	32 (12.8)		
MELD score (median–IQR)	10.04 (7.90–13.63)	1.07 (1.04–1.10)	<0.001 *
NLR (median–IQR)	2.34 (1.5–3.9)	1.12 (1.08–1.15)	<0.001 *
PLR (median–IQR)	94.92 (61.57–139.37)	1.02 (1.01–1.03)	<0.001 *
MLR (median–IQR)	0.40 (0.28–0.59)	1.21 (1.02–1.43)	0.028 *
SIRI (median–IQR)	1.22 (0.74–2.49)	1.07 (1.05–1.09)	<0.001 *
SIII (Log_10_) (median–IQR)	318.2 (160.0–587.3)	2.30 (1.64–3.22)	<0.001 *

BCLC: Barcelona Clinic Liver Cancer; NLR: neutrophil-to-lymphocyte ratio; PLR: platelet-to-lymphocyte ratio; MLR: monocyte-to-lymphocyte ratio; SIRI: Systemic Inflammation Response Index; SIII: Systemic Immune-inflammation Index; IQR: interquartile range. * Statistically significant.

**Table 2:** Individual C-index values, ordered from highest to lowest, for each prognostic variable of overall survival in untreated hepatocellular carcinoma.

Prognostic Factor	C-Index (Standard Error)	CI 95%
BCLC	0.717 (0.017)	0.684–0.750
Albumin	0.713 (0.020)	0.674–0.752
Child–Pugh Score	0.677 (0.018)	0.642–0.712
Charlson score	0.672 (0.019)	0.635–0.709
NLR	0.640 (0.016)	0.609–0.671
Milan criteria	0.639 (0.016)	0.608–0.670
MELD score	0.626 (0.023)	0.581–0.671
PLR	0.605 (0.018)	0.570–0.640
SIII	0.603 (0.018)	0.568–0.638
Age	0.595 (0.022)	0.537–0.623
SIRI	0.593 (0.018)	0.558–0.628
AFP	0.592 (0.025)	0.543–0.641
MLR	0.585 (0.017)	0.552–0.618

## Data Availability

Underlying data not included in this article are available from the corresponding author upon reasonable request in accordance with local and national regulations.

## Abbreviations

The following abbreviations are used in this manuscript:

HCC	Hepatocellular carcinoma
BCLC	Barcelona Clinic Liver Cancer
AFP	Alpha-fetoprotein
MELD	Model for End-Stage Liver Disease
NLR	Neutrophil-to-lymphocyte ratio
PLR	Platelet-to-lymphocyte ratio
MLR	Monocyte-to-lymphocyte ratio
SIRI	Systemic Inflammation Response Index
SIII	Systemic Immune-inflammation Index
TACE	Transcatheter arterial chemoembolization
C-index	Harrell’s concordance index
AIC	Akaike Information Criterion
